# Does Nature and Persistence of Substrate at a Mesohabitat Scale Matter for Chironomidae Assemblages? a Study of Two Perennial Mountain Streams in Patagonia, Argentina

**DOI:** 10.1673/031.012.6801

**Published:** 2012-05-27

**Authors:** Luis Beltrán Epele, María Laura Miserendino, Cecilia Brand

**Affiliations:** CONICET, Laboratorio de Investigaciones en Ecología y Sistemática Animal (LIESA), Universidad Nacional de la Patagonia, Sede Esquel, Sarmiento 849, 9200 Esquel, Chubut, Argentina

**Keywords:** midges, *lsoetes*, *Myriophyllum*, habitat, fluvial

## Abstract

Chironomid substrate—specific associations regarding the nature (organic—inorganic) and stability (stable—unstable) of different habitats were investigated at two low order Patagonian streams, during high and low water periods. Nant y Fall and Glyn rivers were visited twice (October 2007 and March 2008) and seven different habitat types were identified. A total of 60 samples were collected using a Surber sampler (0.09 m ^-2^ and 250 µm) and a set of 23 environmental descriptors including physicochemical parameters and different fractions of particulate organic matter were assessed. 35 Chironomidae taxa were recorded with Orthocladiinae (20), Chironominae (7), and Podonominae (4) being the most well—represented subfamilies. *Paratrichocladius* sp. 1, *Parapsectrocladius* sp. 2, *Parametriocnemus* sp. 1, *Pseudochironomus* sp., and *Rheotanytarsus* sp. were the most abundant taxa. According to the relative preference index, at least 14 taxa showed strong affinity for a particular substrate. The structurally complex macrophyte *Myriophyllum quitense* supported 11 taxa compared with only five taxa found on the less complex *Isoetes savatieri.* Generally, stable substrates (boulders, cobbles, and rooted plants) supported significantly higher chironomids richness, abundance, and diversity than unstable ones (gravel—sand). Canonical correspondence analysis revealed that detritus (leaves, seeds, and biomass), macrophyte biomass, and secondarily hydraulic variables had high explanatory power on chironomids species composition and structure. This work suggests that more complex substrates showing persistence in the temporal dimension supported a diverse array of chironomids, meaning that the maintenance of natural habitat heterogeneity is essential for the community. Land—use practices having significant effects on ecological stream attributes such as increased turbidity, sediment deposition, and runoff patterns will alter assemblages. Understanding environmental associations of the Chironomidae assemblage at the habitat scale is significant for conservation purposes and for the management of low order streams in Patagonia.

## Introduction

Running waters can vary dramatically in size, current velocity, and substrate composition, offering a diverse range of habitats for aquatic insects (Ward 1992). The abiotic habitat template is generally considered to be the major determinant of community organization in fluvial systems, in which physical features of streams create specific habitat conditions supporting different types and forms of food for the aquatic biota (Buss et al. 2004). Spatial and temporal heterogeneity rules the persistence of species in community assemblages (Hynes 1970; Southwood 1977; Ward 1989; Townsend et al. 1997). Consequently, macroinvertebrate assemblage structure in streams is determined by physical factors, such as stream bed geomorphology (Cummins and Lauff 1969; Wallace and Webster 1996), hydraulic features (Statzner et al. 1988), biological interactions (Power 1992; Thomson et al. 2002), and also by the land— use adjacent to the river channel (Resh et al. 1988).

At the reach spatial scale, riverbed morphology shows an alternating sequence of pool and riffle habitats. Pools are slowly flowing depositional zones dominated by fine particles of mineral substrates, while riffles are turbulent, shallower, fast—flowing erosional zones dominated by large particles of mineral substrates (Brussock and Brown 1991; Gordon et al. 1992; Giller and Malmqvist 1998). The contrasting differences in abiotic (i.e., current velocity, substrate composition, water depth) as well as biotic (i.e., food sources, predation) factors between riffles and pools may exert a strong effect on the organization of macroinvertebrate assemblages (Allan 1995; Rabeni et al. 2002). In fact, riffles are generally considered to support higher densities of benthic macroinvertebrates than pools (Hynes 1970; Kerans et al. 1992; Allan 1995; Weigel et al. 2003; Townsend et al. 2004).

The family Chironomidae has a worldwide distribution and is an ecologically important group of aquatic insects, often occurring in high densities and diversity (Merritt and Cummins 1997; Ferrington et al. 2008). They are key members of stream macroinvertebrate assemblages, playing an important role in detritus processing and food chains, since they are consumed by macroinvertebrates and fish (Pinder 1986; Ruse 1992; Armitage et al. 1995; Paggi 2009). Some chironomids are also known to be opportunistic and rapid colonizers adapting to fluctuating conditions (Ladle et al. 1985; Huryn 1990; Ruse 1995).

Furthermore, the high diversity displayed by the group has allowed scientists to use them as bioindicators of water quality (Wilson and Bright 1973). In the last decades chironomids have been used to characterize the status of fluvial systems (Wilson 1979; Laville 1981; Fend and Carter 1995; Cortelezzi et al. 2011).

In Argentina, the composition and distribution of Chironomidae species in rivers and streams and their environmental relationships has been documented in different fluvial systems, including small— to medium—sized mountain streams from the Northwest Andes (Medina and Paggi 2004; Tejerina and Molineri 2007; Scheibler et al. 2008), large subtropical rivers (Marchese and Paggi 2004), and Patagonian reservoirs (Paggi and Rodrígues Capítulo 2002). The use of midge species in biomonitoring was explored in Paggi (2003), and García and Añón Suárez (2007) assessed the structure of Chironomidae community in an Andean Patagonian stream. Nevertheless, the approach about habitat type and Chironomidae species preference has received less attention (Príncipe et al. 2008).

As exposed in recent works, the ecological integrity of river systems in Patagonia is being endangered by land—use changes (Miserendino et al. 2011). Anthropogenic actions can potentially modify habitat suitability (Hall et al. 2001), having dramatic and persistent impacts on biotic assemblages (Niemi et al. 1990). Like other members of aquatic invertebrate fauna in the region, Chironomidae show a high level endemism (Donato et al. 2009). In consequence, the knowledge of Chironomidae community and their environmental relationships at the habitat scale are important for conservation purposes and for the management of low order streams in Patagonia.

The question of how does substrate heterogeneity and midge habitat preferences shape Chironomidae diversity at a given site has been tested by several authors. As stated by Rosa et al. (2011), a high species richness and diversity of midge larvae can be promoted by large environmental heterogeneity. In line with these observations, Principe et al. (2008) found that the highest richness of Chironomidae corresponded to the most complex habitats; those habitats offering the greatest number of niches for species were more preferred.

This research was conducted at two low order streams (Nant y Fall and Glyn, Northwest Patagonia) during high and low water periods with the aims to (1) compare the attributes of chironomid communities in different habitats, (2) determine substrate—specific Chironomidae associations regarding the nature (organic—inorganic) and stability (stable—unstable) of the habitat, and (3) establish environmental variables determining spatial and temporal distribution of taxa at the habitat scale.

## Materials and Methods

### Study area

The study area belongs to the Andean—humid and sub—Andean sub—humid regions (Paruelo et al. 1998), and is located in a transitional mountain and piedmont area in the Northwest of Chubut province, Argentina. From a phytogeographical perspective, the study area is located in the ecotone between the sub— Antarctic forest and the Patagonian steppe, and exhibits a marked altitudinal gradient.

Catchments are dominated by granites and diorite rocks. Extensive outcrops of crystalline bedrock produce ionically dilute waters, a distinctive characteristic of the Andean—Patagonian Cordillera region (Drago and Quirós 1996). The catchment area of Nant y Fall is 161.8 km^2^, and that of Glyn is 21.7 km^2^. The studied streams, Nant y Fall (3^rd^ order) and Glyn (2^nd^ order), are tributaries of the Futaleufú River and Corcovado River, respectively. Nant y Fall dominant land—use is pasture, whereas Glyn is under mixed management, with wood collection (*Nothofagus antarctica* and *N. pumilio*) and extensive livestock (mainly cows and sheep) that is sustained by the herbaceous stratum.

Stream flow in the region is strongly linked to rainfall and snowmelt, which result in predictable winter and spring spates. However, during summer, river flow is low and stable (Coronato and del Valle 1988).

Dominant riparian vegetation at Glyn stream is composed of native *Nothofagus antarctica* (30%), whereas the shrub coverage (40%) is represented by *Berberis buxifolia*, *Schinus patagonica*, *Maytenus chubutensis*, *Ribes cucullatum*, *Ovidia andina*, and *Chusquea culeou.* In the herbaceous strata, *Acaena ovalifolia*, *Fragaria chiloeensis*, *Calceolaria polyrrhiza*, *Prunella vulgaris*, *Verbascum thapsus*, *lupulina*, and *Cerastium arvense* are the most common species. Aquatic vegetation is composed mostly of *Veronica serpyllifolia.* Riparian vegetation at Nant y Fall stream is represented by some specimens of *Nothofagus antarctica*, *Schinus patagonica*, and *Berberis buxifolia.* The dominant strata is herbaceous (60–70%), characterized by *Plantago lanceolata*, *Trifolium repens*, *Cerastium arvense*, *Taraxacum officinale*, and *Rumex acetosella* among others. Aquatic vegetation is composed of *Isoetes savatieri*, *Myriophyllum quitense*, *Limosella australis*, *Ranunculus flagelliformis*, and *Callitriche lechleri*, the subemergent *Lilaeopsis macloviana* and *Mimulus glabratus*, and the emergent macrophytes *Veronica anagallis-aquatica*, *Eleocharis albibracteata*, *Juncus burkartii*, *J. diemii*, and *J. microcephalus.*

### Sampling design and reach characterization

Nant y Fall and Glyn rivers were visited twice: once during high (October 2007) and once during low (March 2008) water periods. Current speed was measured in mid channel (average of three trials) by timing a float as it moved over a distance of 10 m (Gordon et al. 2004). Average depth was estimated from five measurements with a calibrated stick along a transverse profile across the channel. Wet and dry widths (from bank to bank) of the channel were also determined. Discharge (m^3^·s^-1^) was obtained by combining depth, wet width, and current velocity as in Gordon et al. (2004).

On each sampling occasion, the water temperature, pH, specific conductivity (µS_20_ cm^-1^), dissolved oxygen (mg O_2_ L^-1^), oxygen saturation percentage, and total dissolved solids (TDS) were measured with a multi— parameter probe (Hach SensION 156, www.hach.com). In order to perform nutrient analyses, water samples were collected below the water surface, kept at 4 °C and transported to the laboratory. Total nitrogen (TN) and total phosphorus (TP) were determined on unfiltered samples digested with persulphate, whereas nitrate plus nitrite nitrogen (NO_3_ + NO_2_), ammonia (NH_4_), and soluble reactive phosphate (SRP) were analyzed using standard methods (APHA 1994).

Attributes of the riparian vegetation were examined at each site using an adaptation of the QBR index (riparian corridor quality index, Munné et al. 2003) for Patagonian streams: the QBRp (Kutschker et al. 2009).

The total QBRp score ranges from 0 points (extreme degradation) to 100 points (excellent quality, natural riparian forest). Habitat quality was evaluated (HCI: habitat condition index) using the assessment procedure for high gradient streams by Barbour et al. (1999). The HCI scores range from 0 to 200, with a score of 200 points indicating that the river is natural and pristine and in its best possible condition. This index evaluates the ability of the stream's physical habitat to support a given fauna, and also acts as a measure of the spatial heterogeneity of the stream (Castela et al. 2008).

### Habitat selection

Flow type, dominant substrate, depth, and vegetation were used to define each habitat type. Flow types were assessed according to Urbanic et al. (2005) and classified as no perceptible flow (1), smooth flow (2), lateral moving water (3), unbroken standing waves (4), and chute flow (5). Dominant particles of substrate were assessed visually, and depth was measured at each habitat with a calibrate stick. The macrophytes, *Myriophyllum quitense* (Saxifragales: Haloragaceae) and *Isoetes savatieri* (Isoetales: Isoetaceae), were consistently represented both spatially and temporally, and were selected as possible habitats in the design.

A total of seven different habitat types were identified. In depositional areas (pools) three habitat types were sampled: gravel—sand (GS), leaf—pack (LP), and macrophytes (*M*. *quitense*) (M). In riffle areas, two different habitats were sampled: boulder—pebble (BP) and boulder—pebble with filamentous algae (BF). In run areas cobble—pebble (CP) and cobble—pebble with the submerged *I*. *savatieri* (IS) were examined. Habitat GS, BP, and CP were common at both Glyn and Nant y Fall rivers, whereas LP and BF were present at Glyn, and M and IS at Nant y Fall.

### Chironomid and detritus analysis

Three Surber samples (0.09 m^2^, 250 µm pore size) were taken at each habitat in each stream, during the dry and wet seasons, for a total of 60 samples. Samples were fixed with formaldehyde in the field. Individuals and detritus from each sample were sorted in the laboratory.

Sorting involved elutriation and the collection of materials on a series of sieves (mesh width 250–1000 µm). Detritus was divided into fine (250–1000 µm, FPOM) and coarse (> 1000 µm, CPOM) particulate fractions. CPOM was separated into wood, leaves (mainly entire leaves), seeds, and others (fragments of leaves, grass, roots, buds, etc.) (Voelz and Ward 1990). Additionally, macrophyte and bryophyte biomass was obtained from the samples. All fractions were dried (110 °C for four hours) and weighed with electronic balance to ± 0.5 mg.

Larvae were sorted manually under 5× magnification, counted, and preserved in 70% alcohol. In the laboratory, specimens were observed under stereomicroscope, and when necessary, individuals were mounted in glycerin on a microscope slide and examined (Epler 2001; Paggi 2001). Chironomidae larvae were identified to the lowest taxonomic level possible using available keys (Wiederholm 1983; Epler 2001; Paggi 2009). Density was calculated from counts of all the individuals in a sample. Functional feeding groups were assigned by gut analyses and use of available references (Merritt et al. 2008).

### Data analyses

Principal component analysis (PCA) on log (x + 1) transformed data was used to examine variation in physical and chemical parameters between sites and season. PCA is a method of breaking down or partitioning a resemblance matrix into a set of orthogonal axes (linear model). This method used within its intended limits is a valuable procedure to detect structure in the relationships between variables (Ludwing and Reynolds 1988). Variables considered in the analyses are presented in Table 1.

The chironomid assemblages characteristic of each habitat (of both rivers) was determined by the relative preference index (RPI) (Tickner et al. 2000), which is calculated by dividing the abundance of each taxon in a certain habitat by the total abundance of that taxon recorded in all the habitats. The RPI can vary between 0 (no preference for a particular habitat) to 1 (strong affinity for that place).

Canonical correspondence analysis (CCA) was performed to relate changes in species abundance (35 taxa) with environmental descriptors of the habitat assessed (7). The environmental variables considered in the CCA were flow, depth, filamentous algae, bryophytes, macrophytes, CPOM, FPOM, woods, others, leaves, and seeds. All species and environmental data (excepting pH values) were log (x + 1) transformed prior to analyses. An unrestricted random Monte Carlo permutation test with 999 permutations was performed to determine the statistical significance of environmental variables and canonical axes. Where a variable is highly intercorrelated with others, a high inflation factor (> 20) is identified for that variable during initial analysis. These variables were then removed (CPOM and wood) and analysis carried out on the remaining environmental variables (ter Braak and Smilauer 1998). CCA was performed using the statistical package CANOCO version 4.02 (ter Braak and Smilauer 1998).

**Table 1.**
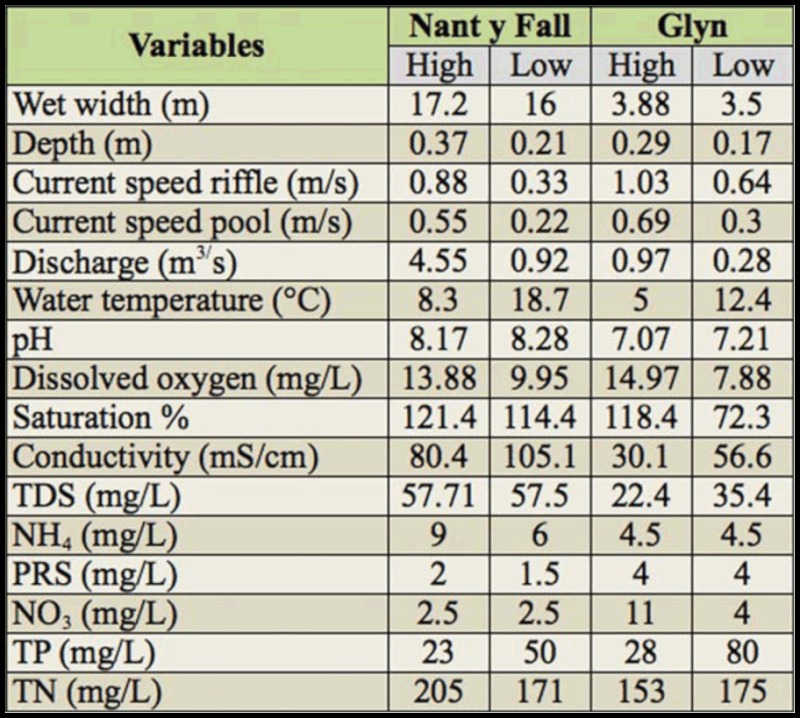
Environmental variables at Nant y Fall and Glyn streams (Patagonia, Argentina), recorded during high and low water periods in October 2007 and March 2008, respectively.

## Results

### Environmental background

Wet width ranged from 16 to 17.2 m at Nant y Fall, and from 3.5 to 3.88 m at Glyn during low and high water periods, respectively. As expected, four physical variables (depth, discharge, current speed in riffle and pool) showed maximum values during the high water periods at both sites (Table 1). Water temperature was ranged from 5 (Glyn) to 18.7 ° C (Nant y Fall). Conductivity and pH values were higher at Nant y Fall than at Glyn. Dissolved oxygen ranged from 7.88 to 14.97 mg·L^-1^ at Glyn stream, and from 9.95 to 13.88 mg·L^-1^ at Nant y Fall.

**Figure 1.**
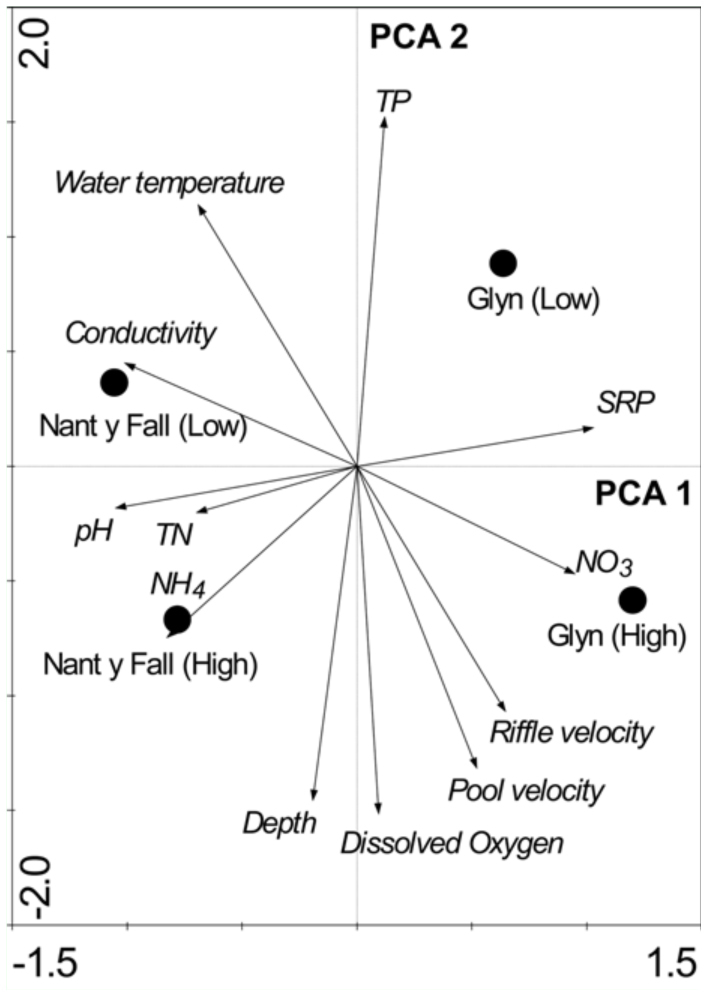
Ordination of sites according to PCA on the environmental variables measured at Nant y Fall and Glyn streams (Patagonia, Argentina) during high (October 2007) and low (March 2008) water period. High quality figures are available online.

Chemical and physical data provided a clear distinction between streams (PCA1) and water period (PCA2) as shown in the PCA ordination graph (Figure 1). The first two factors accounted for most of the variation in the data set (97.9%). The PCA1 (79.7%) highlighted the gradient in chemical conditions of the sites (i.e., high positive loadings by soluble reactive phosphate and nitrate plus nitrite nitrogen; negative loadings by conductivity, pH, and total nitrogen). The PCA2 (18.1%) evidenced a gradient in oxygen content, flow attributes, and level of total phosphorous (i.e., negative loadings by dissolved oxygen, depth, pool and riffle velocity, and high positive loading by total phosphorous).

Riparian corridor quality index for Patagonia (QBRp) values were 62 and 74 for Nant y Fall and Glyn, respectively, indicating that the former had an important riparian ecosystem alteration, whereas Glyn showed incipient disturbance. Regarding habitat conditions the score was 123 (suboptimal) for Nant y Fall and 151 (optimal) for Glyn.

**Table 2.**
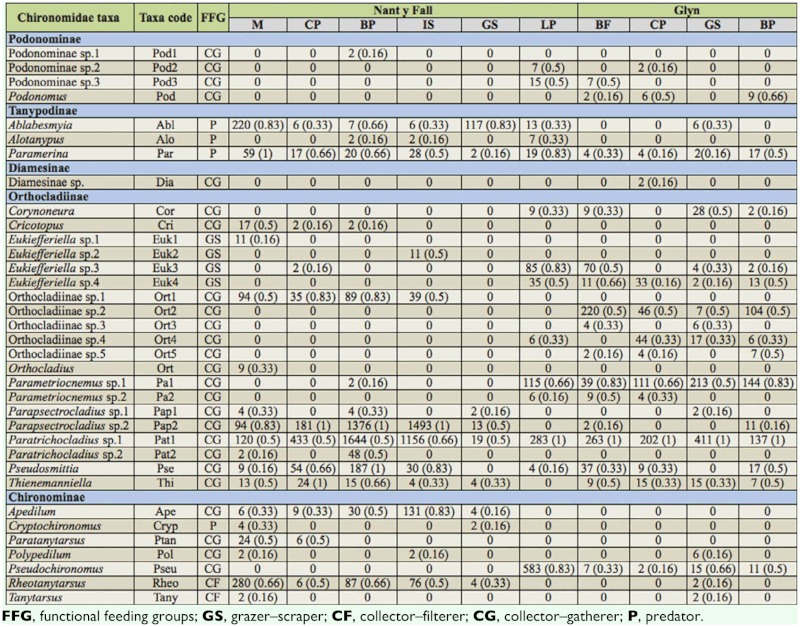
Mean density (ind·m^-2^) and frequency (in brackets) of 35 Chironomidae taxa on different habitat types at Nant y Fall and Glyn streams (Patagonia, Argentina).

**Figure 2.**
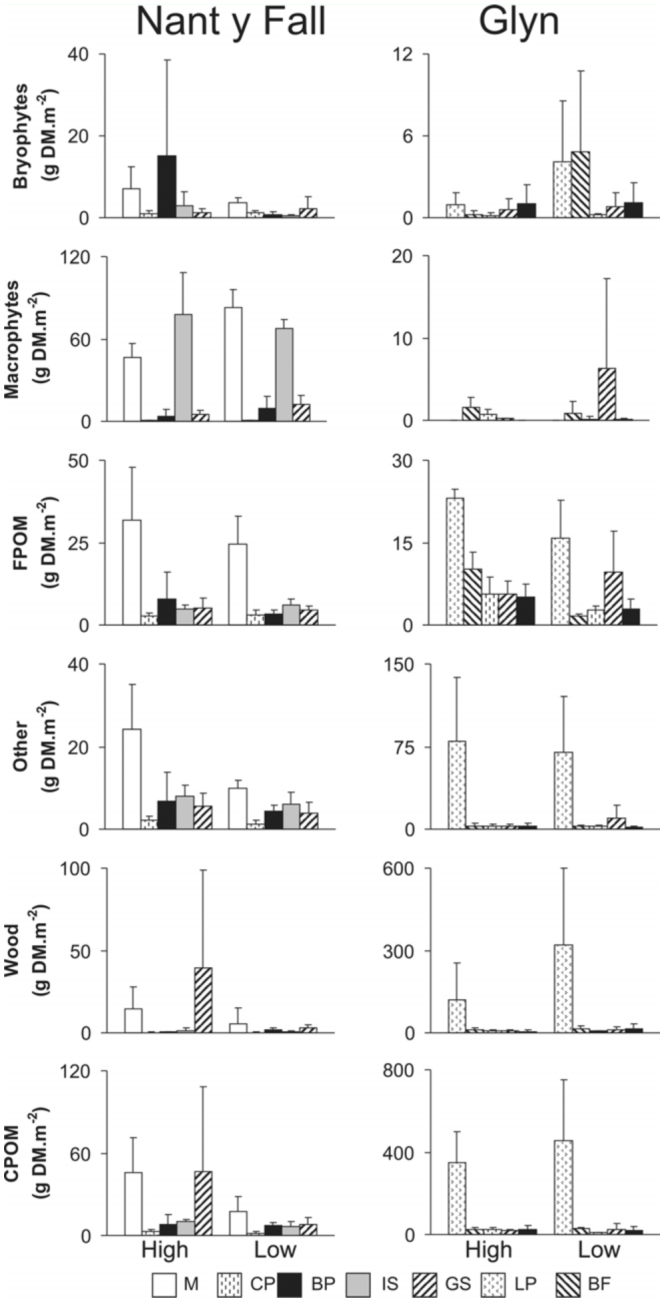
Inter—habitat and seasonal variation in mean total values (g DM·m^-2^) ± SD of organic matter categories (n = 3) per stream (Nant y Fall and Glyn, Patagonia, Argentina). Note the scales on the y—axes differ among streams. Habitats codes: **M**, macrophytes; **CP**, cobble—pebble; **BP**, boulder—pebble; **IS**, *Isoetes*; **GS**, Gravel—sand; **LP**, Leaf—packs; **BF**, boulder—filamentous algae. High quality figures are available online.

The macrophytes item was the best represented at Nant y Fall stream (Figure 2), being dominant at M and IS habitats. The fractions FPOM and others were also represented in M habitats showing maximum values during the high water period. Wood and CPOM items contributed mostly at M and GS habitats. Bryophytes peaked at BP habitats during the high water period (15.1 g DM·m^-2^).

At Glyn stream CPOM and wood were the dominant detrital items at LP habitats (Figure 2), reaching values of 457 and 320 g DM·m ^2^ respectively. Moreover, the fractions others and FPOM peaked at LP habitat during the high water period. During the low water period, bryophytes dominated at BF (5.93 g DM·m^-2^) and LP (4.46 g DM·m^-2^) habitats, whereas macrophytes dominated at GS (6.36 g DM·m^-2^).

At both sites, CP habitat sustained the lowest detritus biomass (12.8 and 38.8 g DM·m^-2^ at Nant y Fall and Glyn, respectively).

### Spatial selectivity of Chironomidae species

35 Chironomidae taxa and morphotypes were recorded in the study, with Orthocladiinae (20), Chironominae (7), and Podonominae (4) as the best represented subfamilies. Total Chironomidae taxa was similar at both sites (23 and 25 for Nant y Fall and Glyn, respectively); however, 12 taxa were exclusively recorded at Glyn stream whereas 10 taxa were found only at Nant y Fall. *Paratrichocladius* sp. 1 was the most frequent and abundant taxa at both sites (Table 2). *Ablabesmyia* sp. and *Parapsectrocladius* sp. 2 contributed mostly at Nant y Fall, while *Parametriocnemus* sp. 1 dominated at Glyn.

**Figure 3.**
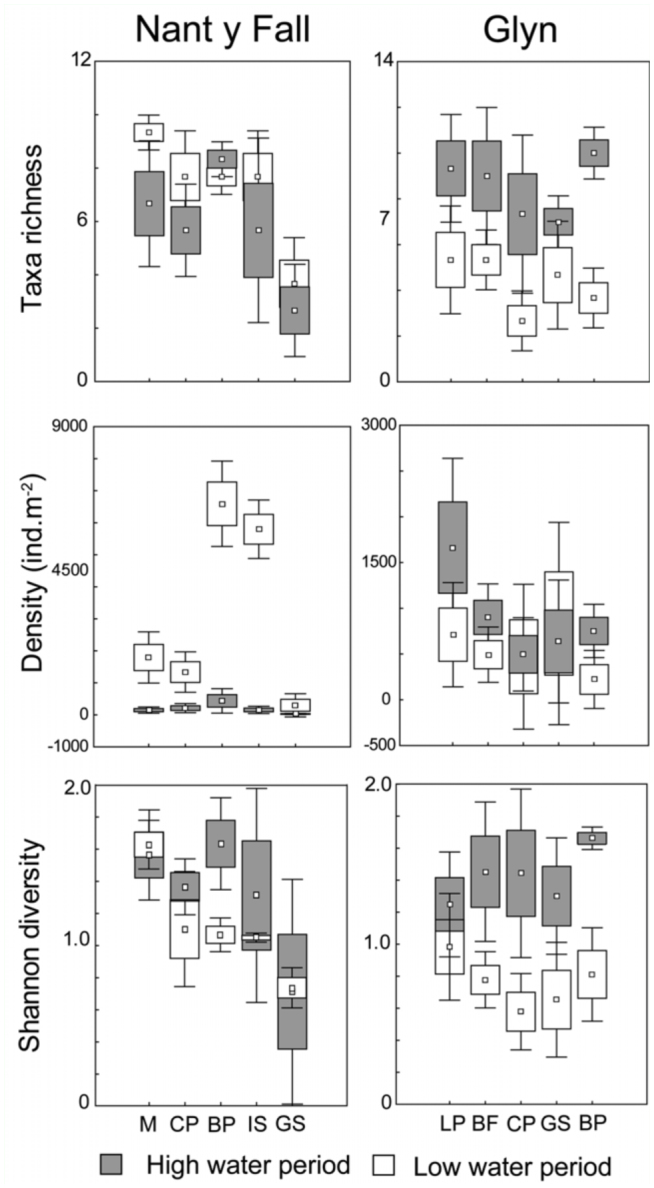
Seasonal values of taxa richness, density (ind·m^-2^), and Shannon diversity of Chironomidae larvae per habitat at two low order streams of Patagonia. Range bars show maxima and minima, boxes are interquartile ranges (25–75%), small squares are means. Habitats codes: **M**, macrophytes; **CP**, cobble—pebble; **BP**, boulder—pebble; **IS**, *Isoetes*; **GS**, Gravel—sand; **LP**, Leaf—packs; **BF**, boulder—filamentous algae. High quality figures are available online.

A comparison on taxa richness, density, and diversity (H) during the low and high water period is presented in Figure 3. Boulder— pebble (BP) sustained significantly higher richness than gravel—sand (GS) at Nant y Fall at both seasons (Kruskal-Wallis *p* < 0.05). No differences per habitat were found at Glyn.

However, richness and Shannon-Weaver index (H) was significantly higher during the high water period at Glyn (Kruskal-Wallis *p* < 0.01).

According to RPI values (> 0.4), at least 11 taxa associated with macrophytes (M) (Nant y Fall), eight taxa with boulder—pebble (BP) (Nant y Fall), and seven taxa with leaf—packs habitats (Glyn) (Table 3). At Nant y Fall *Eukiefferiella* sp. 1, *Orthocladius* sp., and *Tanytarsus* sp. were linked to macrophytes (M) (Table 3), while *Eukiefferiella* sp. 3 was associated with cobble—pebble (CP), *Parametriocnemus* sp. 1 with BP, and *Eukiefferiella* sp. 2 with *Isoetes* habitat.

Taxa with specific habitat association at Glyn stream were: *Alotanypus* sp. with LP, Diamesinae sp. with cobble—pebble habitats, and *Parapsectrocladius* sp. 1, *Polypedilum* sp., *Rheotanytarsus* sp., and *Tanytarsus* sp. with gravel—sand habitats.

**Figure 4.**
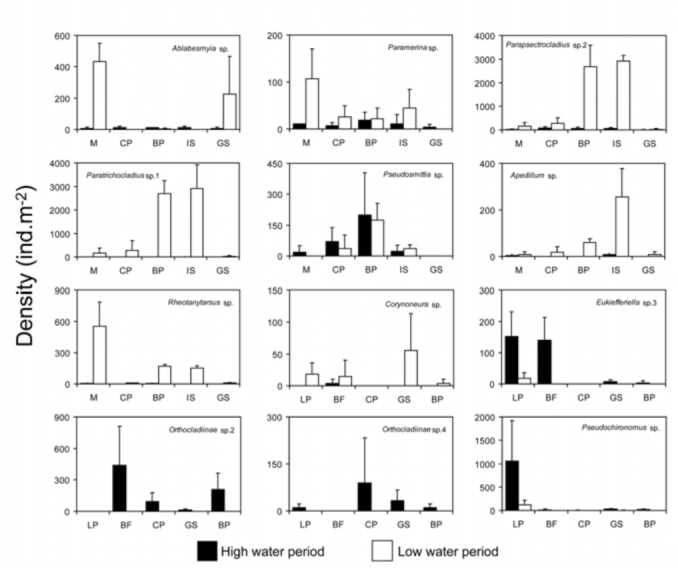
Mean total density (ind·m^-2^) ± SD of 12 chironomids taxa showing habitat preference at studied streams, during high (October 2007) and low (March 2008) water period in Patagonia, Argentina. Habitats codes: **M**, macrophytes; **CP**, cobble—pebble; **BP**, boulder—pebble; **IS**, *Isoetes*; **GS**, Gravel—sand; **LP**, Leaf— packs; **BF**, boulder—filamentous algae. High quality figures are available online.

Regarding temporal patterns of taxa distribution, *Eukiefferiella* sp. 3 (at leaf—packs and boulder with filamentous algae (BF)), *Orthocladiinae* sp. 2 (at BF), *Orthocladiinae* sp. 4 (at cobble—pebble), and *Pseudochironomus* sp. (at leaf—packs) presented high densities during the high water period (Figure 4), and only *Corynoneura* sp. (at gravel—sand) peaked during the low water period. However, at Nant y Fall, most species showed high density values during the low water period. *Ablabesmyia* sp., *Paramerina* sp. and *Rheotanytarsus* sp. showed high densities at macrophytes habitat, whereas both *Parapsectrocladius* sp. 2 and *Paratrichocladius* sp. 1 at boulder—pebble and *Isoetes* habitats, and *Apedilum* sp. at *Isoetes* habitat (Figure 4).

**Table 3.**
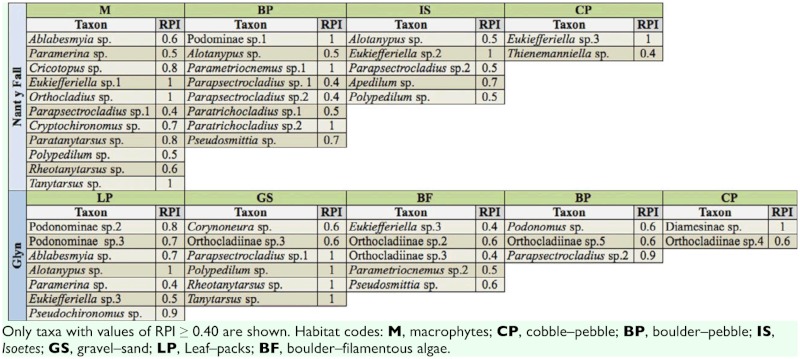
Chironomids associated with each functional habitat and their relative preference index (RPI), for Nant y Fall and Glyn streams.

**Table 4.**
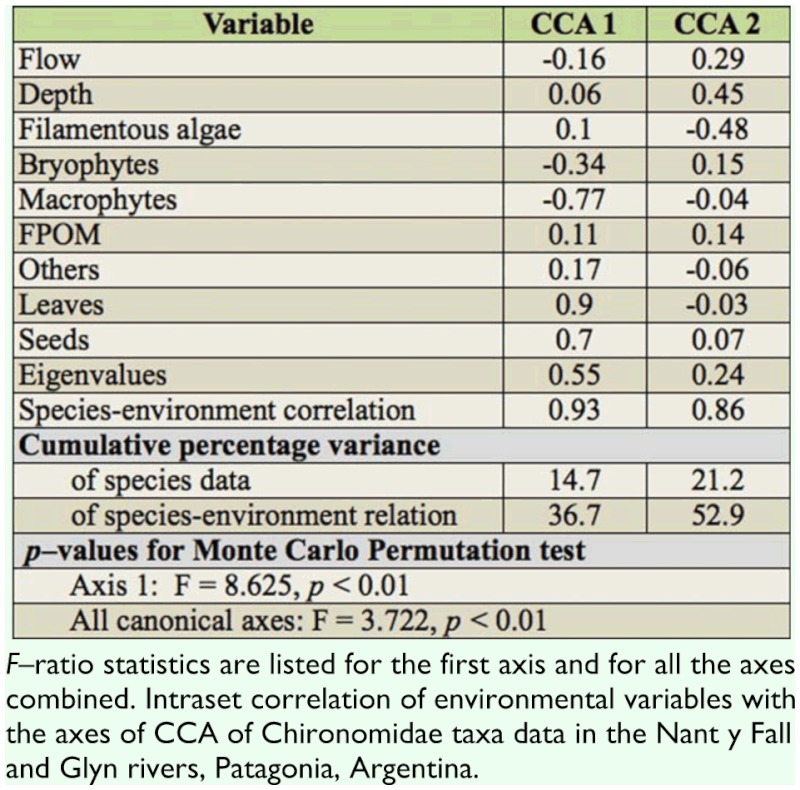
Results of canonical correspondence analysis (CCA). The species—environmental correlations scale the strength of the relationship between species and environment for the axes.

### Chironomidae species and environmental relationships

**Figure 5.**
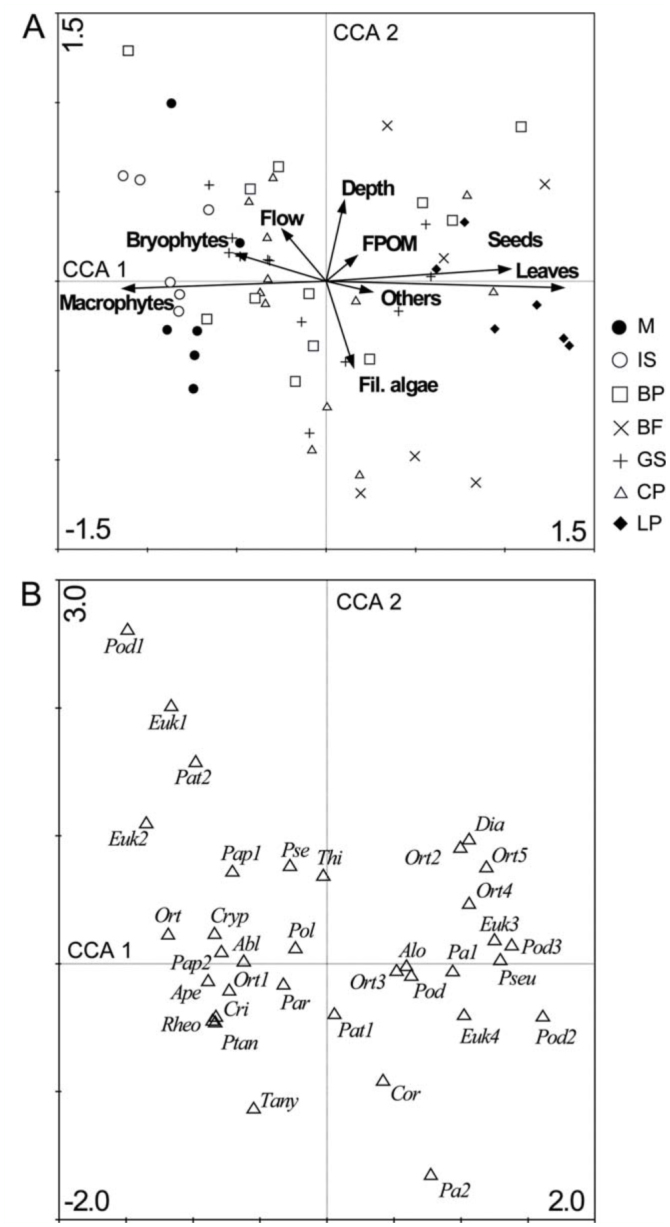
Canonical correspondence analysis ordination plot for sites and environmental variables based on abundance values of chironomids taxa from Nant y Fall and Glyn streams, Patagonia, Argentina. (A) Habitat—environmental biplot; (B) Species distribution according to CCA. Full species names are shown in Table 2. High quality figures are available.

The results of CCA ordination for 35 chironomids taxa, seven habitats, and 13 environmental variables showed that 21.2% of the variance in taxa abundance was accounted for by the first two ordination axes (Table 4). CCA resulted in a significant model as was shown by the Monte Carlo test. The species— environment correlations were 0.93 and 0.87 for the first and second axes, respectively (Table 4), indicating a strong relationship with the environmental variables selected. The first ordination axis (36.7% of the variance of species—environment) reflected a gradient mostly associated with detritus (leaves and seeds), macrophytes, and bryophytes biomass (Figure 5A). Therefore, seed and leaf biomass decreased from the positive to the negative end of the CCA1, whereas bryophyte and macrophyte biomass decreased from the negative toward the positive end of same axis. The CCA2 indicated that stream depth and filamentous algae biomass had the second largest effect on the occurrence of species. Thus, deeper habitats were placed at the positive extreme of CCA2, and shallower habitats at the negative extremes of CCA2.

**Table 5.**
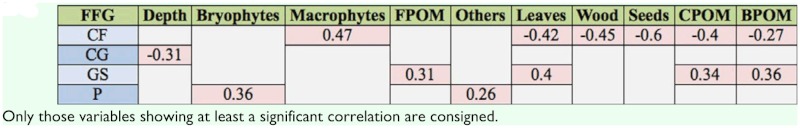
Significant relationships between functional feeding groups (FFG) and environmental variables (R of Spearman's, *p* < 0.05).

Species ordination with regards to the first two axes is presented in Figure 5B. Species occurring in fast flowing habitats with bryophytes (i.e., *Podonominae* sp. 1, *Eukiefferiella* sp.1, *Parapsectrocladius* sp. 2, and *Eukiefferiella* sp. 2) were positioned in the upper left quadrant. Species of slow flowing and shallow waters with filamentous algae were placed in the lower right quadrant (i.e., *Parametriocnemus* sp. 2 and *Corynoneura* sp.). Species mostly associated with leaf—pack (*Eukiefferiella* sp. 3, Podonominae sp. 2 and sp. 3, *Pseudochironomus* sp.) were located toward the positive end of CCA1.

Several significant relationships were observed between functional feeding groups (FFG) and environmental variables (Table 5).

Collector—filterers increased with macrophyte biomass and decreased with depth and biomass of some detrital items (leaves, wood, seeds, CPOM, and BPOM). Grazers—scrapers increased in density with increases of FPOM, leaves, CPOM, and BPOM. Predators showed positive correlation with bryophyte and others biomass.

## Discussion

Chironomid richness observed at our study was comparable to that reported by Príncipe et al. (2008) in upland reaches at Cordoba province (31 taxa), but was significantly higher than that documented by Tejerina and Molineri (2007) for Monte and Yungas (16 taxa), and by Scheibler et al. (2008) in an Andean stream in Uspallata (Mendoza) (seven taxa). Nevertheless, richness values were lower than observed by Garcia and Añón Suárez (2007), which reported 55 taxa for Ñireco stream (at a lower latitude in the Patagonian Mountains). Pupal exuviae were analyzed, which undoubtedly increased the level of taxonomic resolution, and pointed out the need for further taxonomical studies, because richness in Chironomidae inventories from Patagonia often appear low when compared to those in rivers from the Northern Hemisphere (Ruse 1995; García and Laville 2000).

As previously observed in low order streams in the region (Velásquez and Miserendino 2003), Orthocladiinae and Chironominae were the best represented subfamilies in terms of richness and abundance at both sites. Members of these two subfamilies seem to dominate mountain streams in temperate areas in both the Southern (Collier 1993) and Northern Hemispheres (Syrovátka et al. 2009). Orthocladiinae chironomids prefer low water temperatures, which are associated with high latitude and altitude and well— oxygenated conditions (Pinder 1995; Paggi 2009). The cold stenothermic Podonominae and Diamesinae were less abundant in our study, similar to the results of Garcia and Anon Suárez (2007) in a Patagonian mountain stream.

It has been stated that abundance of aquatic macroinvertebrates seems to be considerably higher on aquatic plants than in non—vegetated areas (i.e., Orth et al. 1984; Hemminga and Duarte 2000), increasing with plant density or biomass (Diehl and Kornijów 1998; Attrill et al. 2000). Of the two species of macrophytes studied in the present work (*M*. *quitense* and *I*. *savatieri*), just *I*. *savatieri* sustained significantly higher chironomid density than gravel sand habitats. Concerning chironomid richness, *Myriophyllum* supported higher values than gravel—sand. However, at least 11 chironomid taxa were associated with *M. quitense*, compared with only five taxa found on the less complex *I*. *savatieri*, suggesting that a structurally complex macrophyte sustained an important part of the chironomid community. According to Thomaz et al. (2008), food (basically algae and organic detritus) also may increase with increases in macrophyte complexity, and thus may account for the higher invertebrate richness. A major plant complexity may provide refuges from predation (Warfe and Barmutta 2004). However, plant morphology alone can serve as a behavioral stimulus for certain invertebrate taxa, as shown in an experimental study on *Myriophyllum spicatum*, *Chara baltica*, and *Potamogeton pectinatus* in which the most complex *M. spicatum* was preferred (Hansen et al. 2011).

Stability and persistence facing discharge events are other important factors that should be considered when analyzing invertebrate habitat preferences (Death and Winterbourn 1994). Quinn and Hickey (1990) recognized that the effect of substrate size on invertebrate distribution was a consequence of larger substrates being more stable, and thus accumulating more periphyton and coarse organic matter. In the present work, stable substrates (boulders, cobbles, and rooted plants) supported significantly higher chironomid richness, abundance, and diversity than unstable substrates (gravel sand) in pair comparisons at Nant y Fall (Kruskal Wallis, *p* < 0.05), meaning that habitat persistence was quite relevant in defining chironomid assemblages. As revealed in a previous study, the macrophyte *Myriophyllum* seems to sustain a similar density of invertebrates in depositional areas (pools), as do boulders and cobbles, suggesting it provides a stable substratum and feeding site (Velásquez and Miserendino 2003). Aquatic vegetation certainly plays an important role conditioning distribution of Chironomidae in rivers (Principe et al. 2008), especially in those environments at the time of year when variable, high flows can be expected (Miserendino and Pizzolón 2003).

Temperature and current speed are two important parameters that influence the distribution pattern of Chironomidae larvae (Cranston 1995); these variables also affect the availability of trophic resources and substrate size (Lindegaard and Brodersen 1995; Paggi 2003; Rosa et al. 2011). According to the CCA analysis, detritus (leaves, seed biomass), and macrophyte biomass had high explanatory power on chironomid species composition, meaning that the nature of the substrate and the distribution of trophic resources at a habitat scale were key factors. Hydraulic features such as depth and flow played a secondary role. Syrovátka et al. (2009) found that particulate organic matter in sediments was more important than hydraulic conditions explaining chironomid distribution in a stony bottomed river, and Ruse (1994) noticed the same pattern in gravel habitats of the River Pank. Recently, Rosa et al. (2011) showed that although the nature of a substrate acted as a main factor of chironomid distribution in relation to water flow differences, the presence of natural mechanisms of retention contributed to support similar assemblages in areas of different flows.

More common species can be visualised easily for their abundance or frequency, and they usually respond mainly to conspicuous environmental variables. In the present work, some species were recorded in low numbers and were not abundant in the entire study (e.g., Diamesinae sp., Podonominae sp., *Tanytarsus* sp.). According to Siqueira et al. (2011), rare species may be affected by variables that are difficult to measure, particularly biotic factors. Competence and predation can be factors that drive rare species distribution, and this should be taken into account in future studies.

Giller and Malmqvist (1998) claimed that most benthic taxa are substrate generalists, although many may show a degree of preference for broad substrate categories. According to the present study, the collector filterer *Rheotanytarsus* sp. increased with macrophyte biomass and was strongly associated with *M. quitense.* Passive filter— feeders can benefit from the unimpeded velocities and turbulence transmitted through the open plant stand as those of *Myriophyllum* (Wallace and Merritt 1980). At least two species of *Eukiefferiella* were associated with higher flow velocity, which is coincident with hydraulic condition preferences documented for this genus (Collier 1993; Príncipe et al. 2008). A group of taxa was strongly associated with leaf—pack habitats: the collector—gatherers Podonominae sp. 2, sp. 3, and *Pseudochironomus* sp, and the scraper *Eukiefferiella* sp. 3. This diverse array of trophic groups suggest that these taxa may be using this habitat as refuge more than as a food resource. The smaller body sized *Corynoneura* sp. showed an affinity for gravel—sand habitats (Cranston et al. 1983), whereas *Thienemaniella* sp. appeared as a habitat generalist in agreement with Tejerina and Molineri (2007).

The predators *Ablabesmyia* sp. and *Paramerina* sp. were associated with *M. quitense*; these results are supported by Cremona et al. (2008) and Hansen et al. (2008), who reported that the highest predator abundance occurred on *Myriophyllum* when compared with other habitats. On the other hand, the collector gatherers *Paratrichocladius* sp. 1 and *Parapsectrocladius* sp. 2 co—dominated on *Isoetes* and boulder—pebble habitats. Interestingly, boulder—pebble was the habitat that sustained the highest bryophyte biomass that probably enhanced food availability and space for colonization.

As previously discussed, heterogeneous habitats can potentially offer a great number of niches for invertebrates because they are used as refuges and feeding areas, and decrease the probability of predation. More complex substrates showing persistence in the temporal dimension supported more diverse assemblages of chironomids. Thus, the nature and persistence of mesohabitat were important predictors of composition and abundance of the studied taxa. As expected, the simple and less stable gravel—sand habitat was the least preferred. This phenomenon suggests that an increase in fine sediment inputs could result in severe modifications of habitats and fauna in headwater streams. It has been observed that land—use practices such as forestry and pastoral development increase sedimentation symptoms and severely alter benthos assemblages (Miserendino and Masi 2010; Miserendino et al. 2011), meaning that anthropogenic interventions are resulting in pervasive changes in aquatic ecosystems. Because habitat degradation is increasing rapidly in the region, further work is needed to understand the magnitude of changes. Results given in the present paper may be valuable for conservation purposes and management of Patagonian streams.

## **Abbreviations**

BF: boulder—pebble with filamentous algae
BP: boulder—pebble
CCA: canonical correspondence analysis
CP: cobble—pebble
CPOM: course detritus
FPOM: fine detritus; GS, gravel—sand
GS: QBRp, riparian corridor quality index for Patagonian streams
IS: cobble—pebble with the submerged *I.* *savatieri*
LP: leaf—pack (LP)
M: macrophytes (*M*. *quitense*)
SRP: solid reactive phosphate
TDS: total dissolved solids
TN: total nitrogen
TP: total phosphorus
